# Optimising Embodied Carbon in Axial Tension Piles: A Comparative Study of Concrete, Steel, and Timber Piles Using a Hybrid Genetic Approach

**DOI:** 10.3390/ma18092160

**Published:** 2025-05-07

**Authors:** Kareem Abushama, Will Hawkins, Loizos Pelecanos, Tim Ibell

**Affiliations:** Department of Architecture & Civil Engineering, University of Bath, Bath BA2 7AY, UK

**Keywords:** tension piles, timber piles, concrete piles, steel piles, optimisation, genetic algorithm, embodied carbon

## Abstract

The construction industry is a major contributor to the global climate crisis, prompting increasing interest in minimising the embodied carbon of structures, whether through material production regulations or the optimisation of structural elements. While a wide body of literature addresses the reduction of embodied carbon in superstructures, limited attention has been devoted to the optimisation of foundations, particularly piles. This research introduces a hybrid genetic algorithm optimisation tool designed to minimise the embodied carbon of tension piles in different soil conditions. Six different pile types are analysed: solid and hollow concrete piles, steel pipes, universal column (UC) sections, and timber piles in both square and circular forms. The optimal design parameters for each pile type on undrained clay and loose sand are presented and compared. The results demonstrate the potential for reducing the embodied carbon of tension piles when utilising optimised designs. Finally, a case study involving an 8-metre-high cross-road signpost is presented, illustrating the practical application of the proposed optimisation algorithm for reducing embodied carbon in future designs.

## 1. Introduction

### 1.1. Background

The climate emergency is one of the most pressing challenges globally, with unprecedented environmental change due to excessive greenhouse gas emissions. The built environment is a main contributor to this crisis, being responsible for nearly 37% of global energy-related emissions in 2023 [1]. Material production, energy consumption for on-site machinery, material transportation, and overall construction processes are substantial sources of global carbon emissions [2]. As urbanisation and the demand for infrastructure continue to increase, the construction sector’s contribution to the climate crisis expands accordingly. It is thus essential for the industry to adopt sustainable practices, including innovative design optimisation and the use of low-carbon materials, to mitigate its environmental footprint and contribute to global decarbonisation efforts [3].

Structural optimisation plays a key role in addressing the environmental challenges posed by the construction sector. By employing advanced computational techniques, structural optimisation enables the design of resource-efficient structures without compromising safety or performance. For instance, research has shown that optimising concrete structures can result in significant material savings compared with traditional designs [4,5]. For slab design, the application of structural optimisation has been found to reduce concrete use by as much as 25%, while maintaining structural integrity [6,7]. For concrete beams and columns, structural optimisation is a vital concept in minimising the embodied carbon of structures [8,9]. Moreover, foundations account for a considerable share of any structure and are responsible for a considerable portion of the total embodied carbon in many types of structures. For example, foundations contribute 30% of the total embodied carbon in large-scale water infrastructure projects [10], 24% for a 2 MW onshore wind turbine, and 37% for a 2 MW offshore turbine [11]. Deep foundations can also account for more than 30% of the total embodied carbon and cost in low-rise buildings and lightweight structures such as fences and horizontal installations [12].

In the United Kingdom, the most recent UK FIRES report shows that foundations are responsible for nearly half of all ready-mix concrete consumption, equating to approximately 2.5 MtCO_2_e annually. These figures highlight the urgency of reducing embodied carbon in substructures through design optimisation, efficient use of materials, and critical evaluation of standards and practices.

Therefore, there has been increasing interest in minimising embodied carbon in substructures, including minimising the embodied carbon in the context of retaining structures [13,14], concrete pile design [15,16], slope stability [17,18], and raft foundations [19,20].

**Figure 1:**
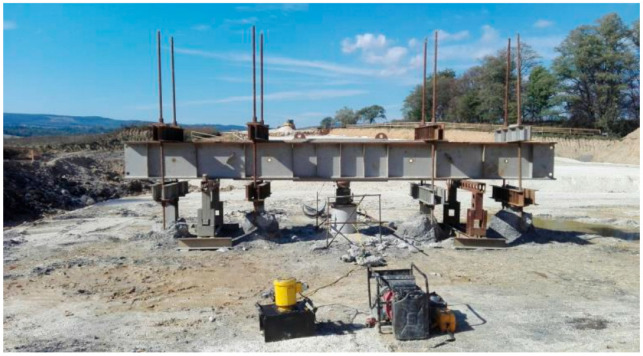
Using tension piles as reaction piles for static loading tests [30].

Tension piles are very common in geotechnical engineering due to their crucial role in ensuring the stability of structures subject to uplift forces and providing resistance to different loading conditions, including wind loads, seismic loads, or even groundwater pressure [21,22]. Their application is particularly important in projects such as resisting uplift forces in high-rise buildings [23,24] and within pile groups for resisting overturning actions in bridges and offshore structures [25,26] as well as supporting static loading tests for compression piles, as shown in Figure 1 [27]. Tension piles can also be used to reinforce soil in areas with weak or unstable soils [28] or in regions prone to environmental stresses such as earthquakes and flooding [29].

Despite the importance of tension piles, very limited attention has been given in the literature to their optimisation. This is believed to be due to the uncertainties surrounding estimating the accurate friction resistance of piles, leading to conservative designs adopting high values of factor of safety and no desire or willingness to achieve optimisation [31,32]. A laboratory investigation involving 31 bored piles, 12 driven piles, and five vibro-driven pullout tests demonstrated that the method of pile installation had a significant influence on the ultimate tensile resistance, and this should be considered when designing tension piles [33]. The degradation of tension pile designs during installation was further studied using a database of 34 pullout pile load tests sourced from the existing geotechnical literature; results showed that a degradation factor should be utilised in an optimal pile design [34]. The degradation of offshore scour-affected tension piles was studied, and a simplified method was developed to account for various scour-hole dimensions around optimised tripod piles in offshore structures [35]. A finite element study on the use of timber piles as tension and bending resisting elements for embankment support showed the effects of GLTP (geosynthetic-reinforced load transfer platform) extent, embankment height, pile spacing, GLTP configuration on the performance of optimised support systems [36]. Metal screw tension piles also stand as a circular alternative to concrete tension piles, and the optimisation of this type of pile has been further addressed in the literature [37]. Nevertheless, no available studies on optimising the environmental impact of tension piles have been recorded, a gap that is addressed in this research.

### 1.2. Aims and Objectives

The authors have previously addressed optimising the embodied carbon in piles subjected to axial compression [16], lateral forces [38] as well as investigating the embodied carbon from different piling materials [39]. Results showed a strong influence of geometry and design parameters on the environmental impact of piles, suggesting potential room for improvement. Moreover, it was shown that, for piles subjected to pure axial compression, timber piles are the most environmentally-friendly option but are only suitable at low capacity (<1.3 MN). Hollow concrete piles were also shown to be significantly more carbon-efficient than conventional solid concrete piles, in all soil types.

However, no attention has been given to piles subjected to pure tensile stresses. Therefore, this research aimed to discover the optimal concrete, steel, and timber pile designs under pure tensile loads in both undrained clay and loose sand through the following objectives:

1. Introduce a robust optimisation algorithm that is capable of producing pile designs with the lowest embodied carbon for different soil conditions and pile capacities.
2. Deploy the optimisation algorithm to discover the optimal design of six different pile types; concrete solid, concrete hollow, steel pipe, steel universal column (UC) sections, timber circular, and timber square, in a range of common soil types.
3. Compare the characteristics of optimal tension piles and optimal compression piles, in order to provide generalised design guidance.
4. Apply the optimisation algorithm to an existing case study to assess the potential carbon savings for a built structure, to support future endeavours.

### 1.3. Analysis Setup

This research was structured into four sections, to address the four main research objectives as follows:

(a) Deploy the optimisation algorithm to produce optimised tension piles in undrained clay soil: Concrete, steel, and timber piles with capacities up to 3 MN were designed for use in undrained clay conditions. The optimal design parameters for each material type were determined and compared.
(b) Deploy the optimisation algorithm to produce optimised tension piles in loose sand: Concrete, steel, and timber piles were designed for capacities up to 3 MN in loose sand conditions, and the optimal design parameters were compared.
(c) Comparative analysis: A broad discussion is provided comparing the optimal design options for compression and tension piles in different soil types.
(d) Case study: A real-world case study of an existing tension pile design is presented in detail. The parameters from the built piles were fed into the optimisation algorithm to generate an alternative optimal pile design.

## 2. Methodology

### 2.1. Pile Capacities and Soil Types

The functional unit in this study is defined as a single pile capable of resisting a specified axial tensile load (e.g., 1.0 MN or 3.0 MN) while satisfying all structural and geotechnical safety requirements. This ensures a consistent basis for comparing the embodied carbon of different materials and geometries. The six tested pile types comprised different materials, geometries, and specifications as shown in Section 2.3.

A safe pile design should at least be capable of satisfying the following two design criteria:

- Structural capacity: the pile resistance as a structural element subjected to pure tensile stresses is within a safe limit.
- Geotechnical capacity: the factored pile’s frictional resistance is less than the applied tensile load.

For this study, a range of tensile pile capacities of up to 3 MN was tested. The following is a summary of the theoretical models used to assess each pile capacity.

#### 2.1.1. Structural Capacity

For piles subjected to tensile forces, the structural capacity is governed by the pile’s ability to resist direct axial tension. In the case of reinforced concrete piles, this capacity is predominantly carried by the longitudinal reinforcement bars, as the concrete is assumed to crack under tension and hence does not contribute significantly to tensile strength. Therefore, the tensile resistance of concrete piles is computed based solely on the steel reinforcement area and its yield strength, as per Eurocode 2 and as shown in Equation (1) [40]. For steel piles and timber piles, the full cross-sectional area contributes to the tensile capacity, and their capacities are evaluated using Eurocode 3 and Eurocode 5 and as shown in Equations (2) and (3), respectively [41,42]. This distinction is important to guide readers on how each material behaves differently under tensile loading.

(1) $$N_{con.}=\frac{{\;f}_{y}}{\gamma_{p}}\;\cdot \;A_{s}$$

(2) $$N_{stl.}=\frac{f_{y}}{\gamma_{p}}\;\cdot \;\;A_{s}$$

(3) $$N_{tim.}=n\;\cdot \;\frac{f_{ctk}}{\gamma_{m}}\;\cdot \;A_{t}$$

where the following apply:

- $N_{con.},\;N_{stl.}\;\mathrm{and}\;N_{tim.}$ = concrete tensile capacity, steel tensile capacity, and timber tensile capacity.
- $A_{s}$ and $A_{t}$ = steel cross-sectional area and timber cross-sectional area
- $f_{ctk}\;\mathrm{and}\;f_{y}$ = characteristic compressive strength of timber and steel.
- $\gamma_{m}$ = partial factor for timber strength = 1.3 [43]
- $\gamma_{p}$ = partial factor for steel strength = 1.3 [43]
- *n* = reduction factor for timber class 3, submerged in water = 0.8 [42]

#### 2.1.2. Geotechnical Capacity

**Figure 2:**
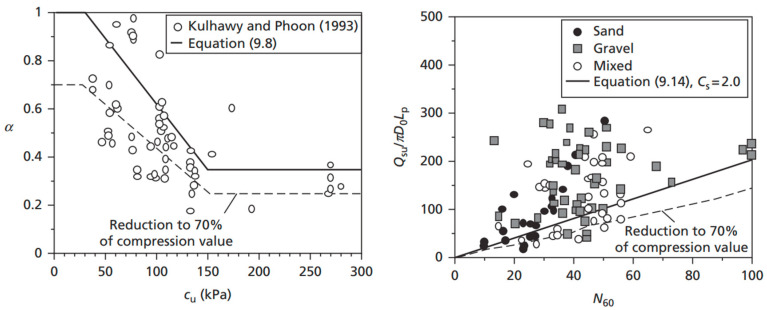
Validating De Nicola and Randolph’s [46] proposed tensile capacity ratio against the published literature [47,48] as reported by Knappet and Craig [49].

There exist several methods for assessing the tension capacity of piles (*Q_t_*), including but not limited to the Alawneh method, which was a result of 34 pull-out pile load tests collected from the literature [34]. The American Petroleum Institute (API) method was based on field experiments performed on instrumented closed-ended displacement piles [44]. The ICP-05 design method was based on results from load tests on jacked closed-ended instrumented piles and was calibrated for open-ended piles primarily using tests on driven piles [45]. However, one of the most commonly used and simply interpreted methods for estimating the tensile capacity of piles was proposed by De Nicola and Randolph [46]. Based on experimentation, the authors were able to calculate the tensile pile capacity (*Q_t_*) as a ratio of its surface friction resistance, under compressive loading (*Q_c_*) as shown in Equation (4). Figure 2 presents a validation of the tensile-to-compressive capacity ratio model proposed [46], as expressed in Equation (4). The plotted data were extracted from the experimental results reported by Kulhawy and Phoon [47,48] as well as other validated studies compiled by Knappet and Craig [49] and were found to be representative of the actual tensile capacities of the piles. These datasets represent measured values of axial tensile and compressive resistances of piles in both fine and coarse soils.

The plotted trendline, based on Equation (4), provides a reliable lower-bound estimate for tensile capacity relative to compressive capacity across a range of pile geometries and soil conditions. This supports the adoption of Equation (4) in the proposed model for predicting conservative but realistic tension pile capacities. Additionally, Equations (5)–(7) are used to determine the compressive resistance based on shaft friction, which is then converted into tensile capacity using the validated ratio.

(4) $$\frac{Q_{t}}{Q_{c}}\approx [1-0.2\log_{10}\left(\frac{100D}{L}\right)][1-8(\frac{v\cdot L\cdot G}{D\cdot E})+25{\left(\frac{v\cdot L\cdot G}{D\cdot E}\right)}^{2}]$$

(5) $$Q_{c=}2\pi \int_{0}^{L}L\;\cdot \;r_{i}\;\cdot \;\tau \;d\left(L\right)$$

(6) $$\tau_{sand}=k_{s}\cdot k_{t}\;\cdot \;\sigma_{v}^{\prime }\;\cdot \;tan\delta$$

(7) $$\tau_{clay}=\alpha \;\cdot \;c_{u}$$

where the following apply:

- L, D, and $r_{i}$ = the pile’s length, diameter, and radius.
- v, *G*, and *E* = Poisson’s ratio, shear modulus of elasticity, and Young’s modulus of elasticity.
- $\sigma_{v}^{\prime }$ and δ = soil effective stress and pile–soil friction angle.
- $\tau_{sand}\;and\;\tau_{clay}$ = the average shear stress (shaft friction) mobilised along the pile shaft in sand and clay soils.
- $k_{s}\;\mathrm{and}\;k_{t}$ = soil and pile friction coefficients.
- *α* = adhesion factor for undrained clay.

#### 2.1.3. Tested Soil Types

In this study, two distinct soil types were examined to investigate potential variations in the optimisation outcomes to capture a broad spectrum of geotechnical behaviours, enabling a comprehensive assessment of how different soil characteristics influence the optimisation process. The first soil type was an undrained clay with a high water table, while the second was dry, loose sand. Both soil models were previously tested in the literature [49], with the relevant parameters summarised in Table 1. The choice of these two contrasting soil conditions aimed to incorporate both saturated clay and dry sand; this investigation seeks to ensure that the findings apply to a wider range of practical scenarios, enhancing the robustness of the conclusions drawn from the optimisation results.

### 2.2. Embodied Carbon Model

#### 2.2.1. LCA Approach

There exist several approaches to calculating the embodied carbon in structures, including but not limited to the Athena Impact Estimator for Buildings [50] and RICS Whole Life Carbon Assessment for The Built Environment [51]; an interactive, easy-to-implement lifecycle assessment method was presented by the Institution of Structural Engineers (IStructE, London, UK) [52]. The embodied carbon calculation method provides a comprehensive framework for assessing the carbon footprint of structural elements, including foundations. Based on lifecycle stages outlined in BS EN 15978:2011 [53], the method covers all stages of a structure’s life, from material extraction and production (A1–A3), through construction (A4–A5), use (B1–B7) and end-of-life processes (C1–C4), to any potential benefits beyond the system boundary (D).

**Figure 3:**
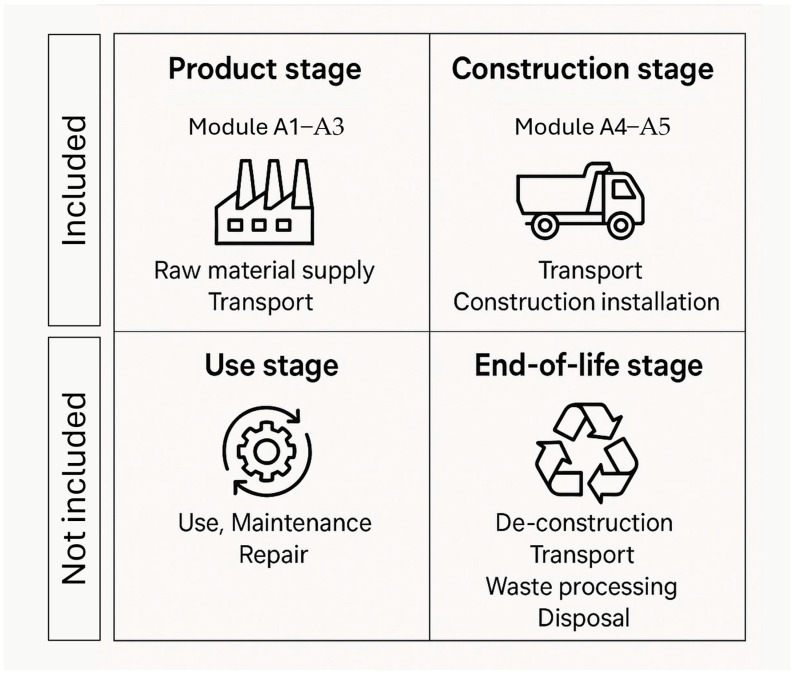
Lifecycle stages included in the proposed LCA model.

In this study, only the lifecycle stages A1–A5 are considered, encompassing material extraction, production, transport to site, and construction-related processes, as shown in Figure 3. It should be noted that in practice, substructure elements such as piles are rarely removed, reused, or recycled. Once installed, piles are typically abandoned in place at the end of their service life, either due to inaccessibility or because their removal offers no environmental or economic advantage. Therefore, modules C1–C4 (end-of-life) and D (beyond the system boundary) are excluded from this analysis. The results are intended to reflect the embodied carbon impact of design decisions made prior to installation. Designers are encouraged to adapt this methodology for different boundary assumptions where applicable, such as for modular or replaceable foundation systems.

This method requires estimation of material quantities, typically drawn from design specifications or actual project data. It involves multiplying these material quantities by corresponding embodied carbon factors and summing the emissions across all lifecycle stages, as shown in Equation (8). In this research, the lifecycle stages A1–A5, which involve embodied carbon during extraction, manufacturing, transportation, and construction, including waste, are considered in the calculations, as piles are rarely refurbished, deconstructed, or recycled.

(8) $$TEC=\sum \left(m_{x}\right)\left({ECF}_{x}\right)\;$$

where the following apply:

- *TEC* = total embodied carbon (kgCO_2_e)
- $m_{x}$ = mass of the construction material (kg)
- ${ECF}_{x}$ = embodied carbon factor for a given material (kgCO_2_e/kg), as shown in Table 2.

#### 2.2.2. Embodied Carbon Factors

Embodied carbon factors provide an estimate of the contribution each unit of material makes to the total embodied carbon of a completed structure. A comprehensive inventory of embodied carbon factor values for a wide range of materials was developed by Craig Jones and Geoffrey Hammond at the University of Bath in 2008 [54]. It is important to acknowledge that these values are project-specific and can vary depending on the materials’ composition and the location of the construction. As such, it is recommended that designers consistently seek accurate embodied carbon factor values from their supply chains [52]. For this study, the values of the embodied carbon factors used for each material and lifecycle stage are presented in Table 2 with references; these are intended to represent generic, industry average values. The embodied carbon factors (A1–A5) used in this study represent UK average values. It is important to note that these values are region- and product-specific and may vary significantly in other contexts. For example, the embodied carbon of steel products depends on the proportion of recycled content (scrap) and the production route (e.g., electric arc furnace vs. basic oxygen furnace), as well as on the product type (e.g., open section, hollow section, or reinforcement bar). The concrete factors similarly vary depending on mix design, cement content, and supplementary cementitious materials (SCMs).

While the results presented here are representative of typical UK practice, they should not be generalised to all settings. Designers applying this method elsewhere should adjust carbon factors to reflect local sourcing, manufacturing, and certification routes. A detailed analysis of the effect of concrete and steel recycled content on the embodied carbon in piles was previously presented by the authors in a separate work [15].

### 2.3. The Optimisation Algorithm

#### 2.3.1. Algorithm Definition

Genetic algorithms (GAs) offer an effective optimisation technique that can be used to solve complex structural engineering problems by mimicking the process of natural selection. They are particularly valuable for structural optimisation, which includes optimising structural layout [55,56], cost [57,58], materials usage [59], and pile optimisation [60,61]. In GA-based optimisation, potential solutions are encoded as “chromosomes” that evolve over generations through selection, crossover, and mutation operations. This approach allows the exploration of a large solution space to identify optimal solutions efficiently.

**Figure 4:**
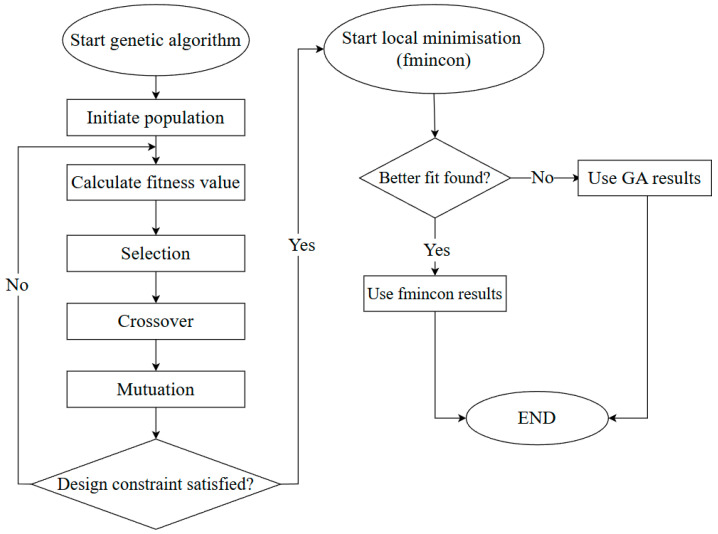
Simplified schematic of the optimisation algorithm.

For this research, a hybrid genetic algorithm was employed to minimise the embodied carbon in tension pile designs, as shown in Figure 4. Genetic algorithms are particularly well suited for navigating complex, multi-dimensional design spaces, but they do not guarantee convergence to a global optimum [61]. Therefore, and to enhance precision and avoid premature convergence, a two-stage approach was adopted as follows:

- In the first stage, the GA explored the full design space using a large initial population (500 individuals) and standard genetic operators (uniform selection, arithmetic crossover, Gaussian mutation). The design space was defined by various design variables with bounds shown in Table 3, corresponding to a multi-dimensional design space with approximately 10^6^ potential design combinations. Range partitions were not discretised a priori but sampled continuously within the GA framework.
- Once the GA converged to a promising solution after 50 stall generations, a refined local search was applied using MATLAB’s fmincon solver, which performed constrained nonlinear optimisation. This step improved the local accuracy of the solution by fine-tuning within a narrower design range around the best GA result.

This approach enhanced the global search capability of the GA while ensuring high local precision through gradient-based refinement.

#### 2.3.2. Section Constraints

**Figure 5:**
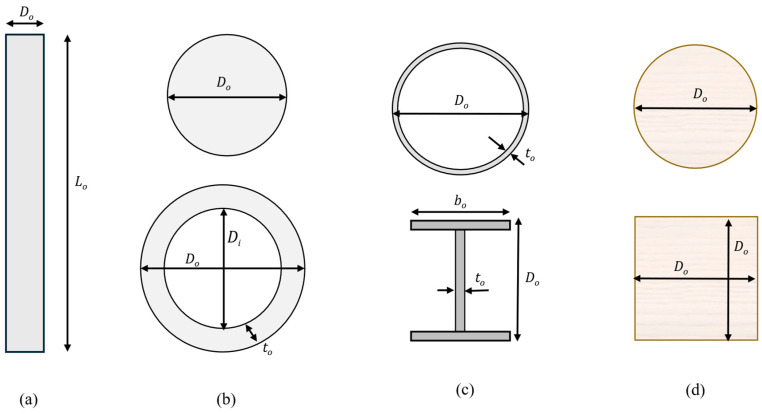
Optimised pile parameters: (**a**) pile elevation; (**b**) concrete cross sections; (**c**) steel cross sections; (**d**) timber cross sections.

Six tested pile types were tested, as shown in Figure 5 and detailed in Table 3, covering various material and shape configurations, including solid and hollow concrete bored piles, square and circular timber piles, steel pipes, and universal column sections. The allowable range of pile lengths varied from 1 m to 300 m to cover the full range of optimal solutions. For concrete piles, diameters ranging between 0.1 to 2 metres and concrete thicknesses of 0.1 up to half the pile diameter were allowed for hollow concrete piles. Timber piles, available in square and circular sections, have lengths from 1 to 12 metres but can be extended using mechanical couplers and diameters or widths from 0.05 to 0.45 metres [63], using Douglas fir C24 timber with a characteristic tensile strength of 18 MPa. Steel pipe and universal column piles were also assessed, with lengths between 1 and 300 m, as were universal sections as provided by UK steel suppliers [64]. The material properties used in this study were selected based on typical values used in UK practice for standard piling systems. For steel piles, a structural steel grade of S355 (yield strength = 355 MPa) was adopted, which is the most common grade used for pile fabrication in the UK and Europe. Reinforced concrete piles were designed using concrete grades ranging from C18 to C60, in accordance with Eurocode 2, and typical reinforcement grades (B500B). For timber piles, C24 Douglas fir was chosen, as this is widely available in the UK and complies with Eurocode 5 for softwood construction.

It is acknowledged that the environmental impact and structural capacity of piles vary with different material grades. However, the chosen grades reflect current industry norms and provide a fair basis for comparison. The optimisation results should therefore be interpreted as indicative for standard solutions, and this approach can be adapted to reflect other material grades or national practices. A discussion of how optimal solutions compare to typical “off-the-shelf” standard designs is included in the case study in Section 4.

For each pile design, the total material mass was calculated based on the optimised geometric dimensions (length and cross section) and standard material densities (2400 kg/m^3^ for concrete, 7850 kg/m^3^ for steel, 500 kg/m^3^ for timber). Embodied carbon was then derived by multiplying these masses by the corresponding emission factors from Table 2. While pile geometry (e.g., L/D ratio) governs structural capacity and stiffness, embodied carbon is governed by material mass.

## 3. Results and Discussion

All pile designs presented in this study were optimised to provide the same axial tensile resistance (e.g., 1.0 MN), ensuring that they were functionally equivalent. Each design satisfied both structural and geotechnical safety requirements, as defined by the relevant Eurocodes (EC2, EC3, EC5), and all designs met standard safety factors and material utilisation limits. It is important to emphasise that the goal of this comparison was not to maximise capacity, but to minimise embodied carbon for a fixed performance level. The variation in embodied carbon is therefore a direct result of the material mass required to meet the functional load.

### 3.1. Undrained Clay Soil

**Figure 6:**
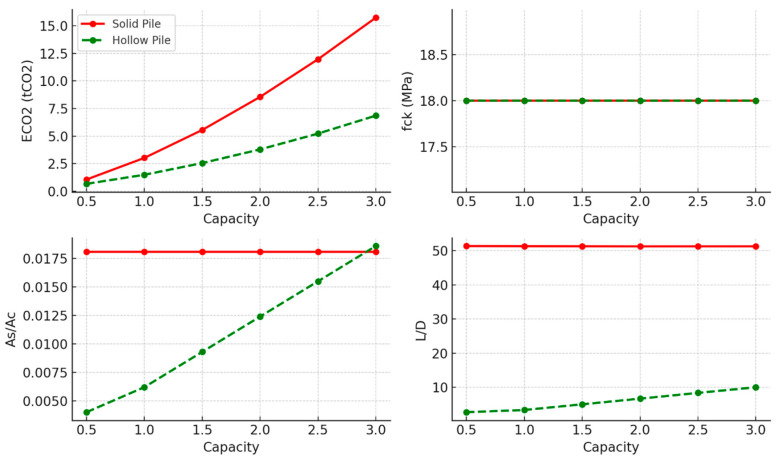
Optimal concrete design parameters for tension piles in undrained clay soil.

The results of the optimised concrete piles in undrained clay soil are shown in Figure 6 and Table 4. They indicate a clear distinction between the performances of the solid and hollow pile designs. Hollow piles demonstrated lower embodied carbon values across the entire tested capacity range, showcasing their material efficiency and sustainability potential. However, this advantage was associated with a rising optimal steel-to-concrete ratio (*As/Ac*). In contrast, solid piles exhibited a notably higher slenderness ratio (L/D), indicating that they may be less efficient in terms of developing surface friction. Moreover, all pile designs favoured a consistent lower-bound compressive strength (*f_ck_* = 18 MPa) across capacities, implying that the concrete strength did not govern the observed variations in embodied carbon or *L/D* ratios, the design was governed by the geotechnical capacity of the pile and the reinforcement steel deployed, and that a minimal concrete grade is needed to build a pile body with sufficient surface friction resistance and steel cover.

**Figure 7:**
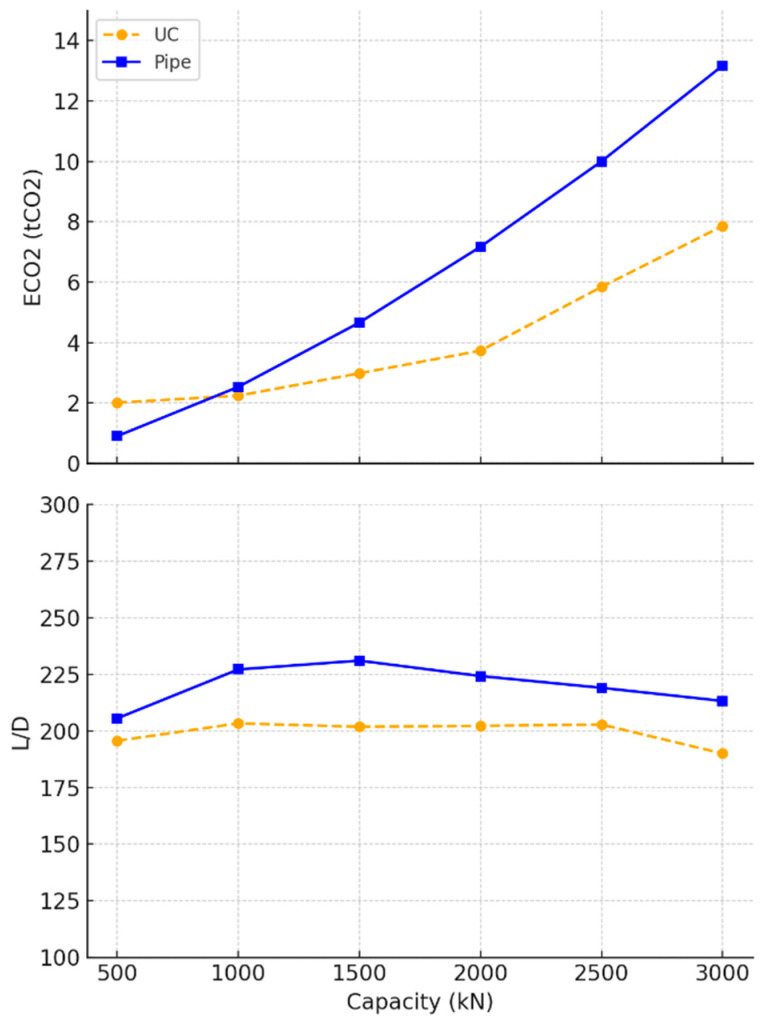
Optimal steel design parameters for tension piles in undrained clay soil.

Figure 7 compares the performance of universal column (UC) and steel pipe piles. The steel pipes demonstrated a steep increase in embodied carbon beyond 1MN, making them less environmentally efficient at higher capacities. In contrast, the UC piles managed to maintain lower embodied carbon levels across the range, indicating that they may offer a more sustainable option for larger loads. However, this was offset by a more pronounced peak in the slenderness ratio (*L/D)*, where UC piles seemed to require greater length-to-diameter proportions before stabilising, as shown in Table 5. The pipes, by contrast, exhibited a gradual decline in their *L/D* ratio. It also should be noted that the inconsistency in the UC data was due to approximations to the nearest universal column sections provided by the manufacturers [64].

**Figure 8:**
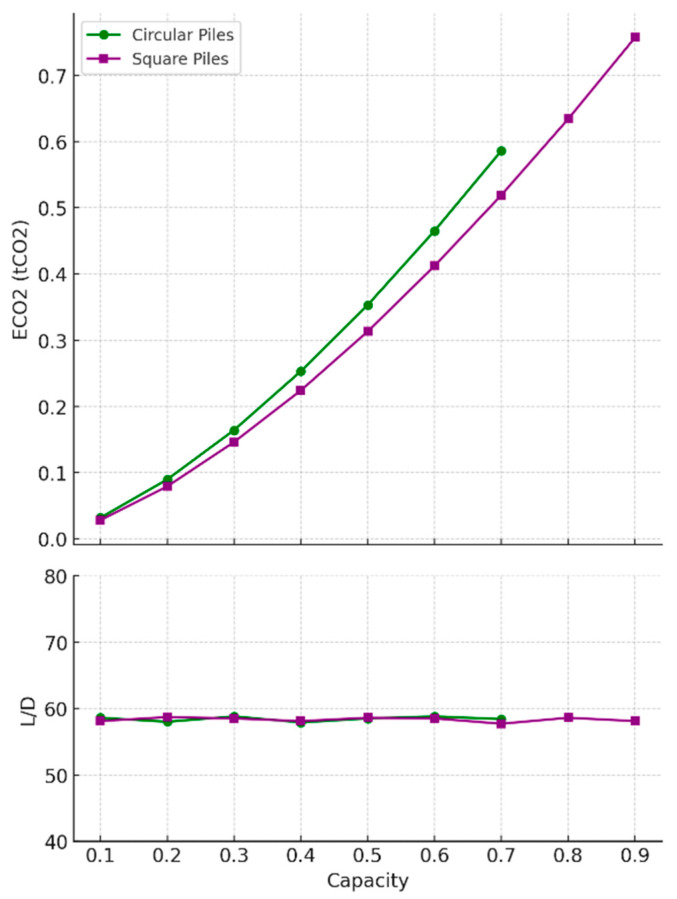
Optimal timber design parameters for tension piles in undrained clay soil.

The results for timber pile optimisation are shown in Figure 8 and Table 6 and reveal that timber piles are only available for low pile capacities of less than 1 MN, due to dimensional constraints. There were slight differences between the circular and square pile designs. Square piles consistently showed slightly lower embodied carbon across different capacities, because of their slightly larger surface area to volume ratio, indicating better environmental impact. Both circular and square piles maintained relatively consistent *L/D* ratios, suggesting that geometric shape had minimal influence on their length-to-diameter efficiency and ensuring that the design was governed by the pile’s structural capacity (tensile strength of timber).

### 3.2. Sand Soil

**Figure 9:**
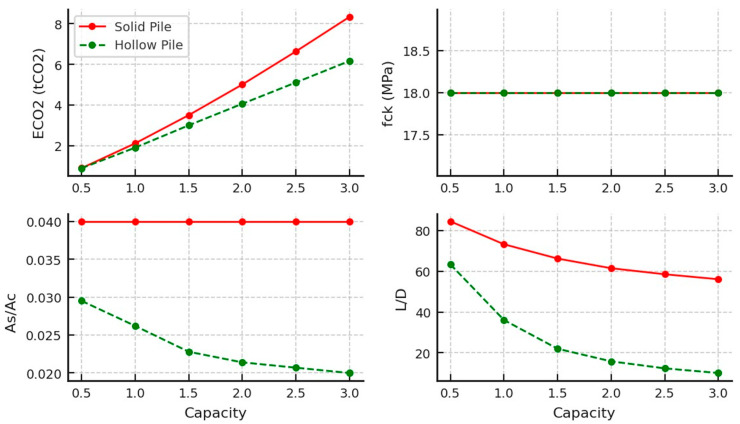
Optimal concrete design parameters for tension piles in loose sand soil.

The optimal designs for concrete piles in loose sand are shown in Figure 9 and Table 4; the performance of solid and hollow concrete piles diverged similarly to the results from undrained clay, though with some nuances. Hollow piles continued to show lower EC values across the capacity range. Unlike in undrained clay, the *L/D* ratio for both pile geometries in loose sand decreased steeply as capacity increased, indicating that the solid and hollow piles both required shorter lengths relative to their diameter to sustain the same loads in loose sand. The compressive strength (*f_ck_*) remained consistent at 18 MPa, suggesting that, again, concrete strength had no effect, as loose sand required a design that maximised surface area for frictional resistance while utilising the lowest possible concrete grade.

**Figure 10:**
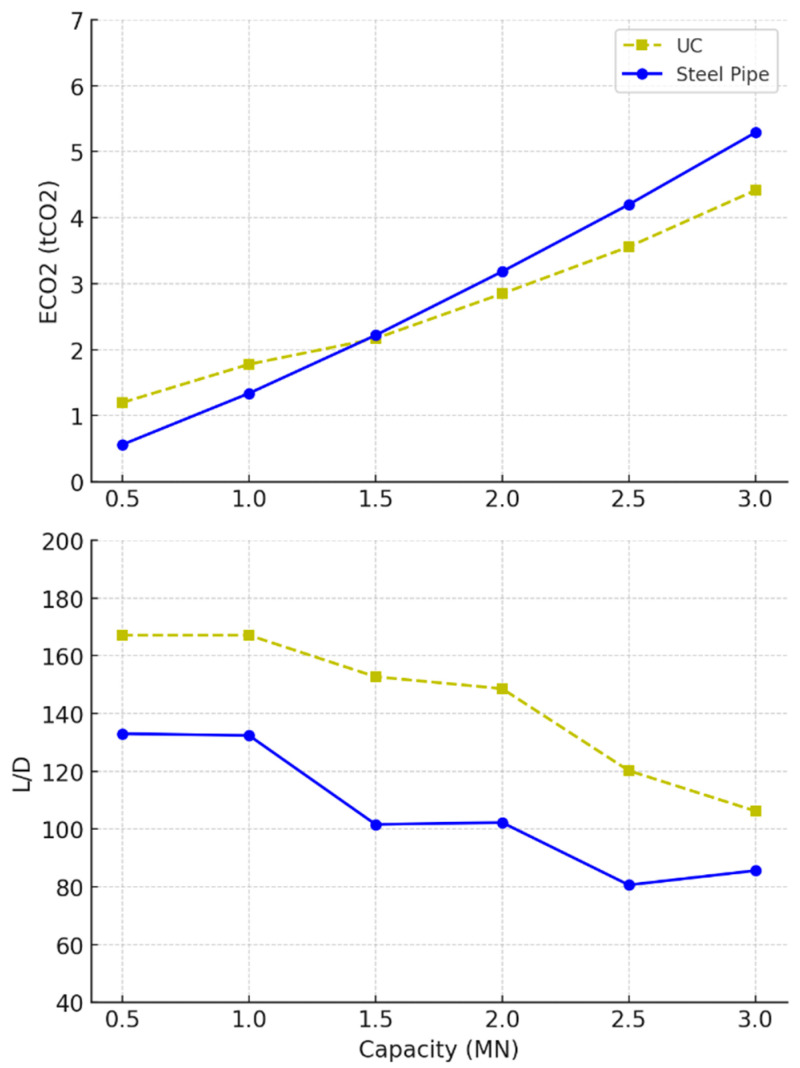
Optimal steel design parameters for tension piles in loose sand soil.

The results for steel piles in loose sand are shown in Figure 10 and Table 5. There was a consistent rise in embodied carbon for both the universal column and pipe designs; however, in contrast to the undrained clay results, the pipes in loose sand were associated with less embodied carbon than the UCs. The *L/D* ratio for steel sections in sand also behaved differently from undrained clay soil; a continued decrease in the optimal slenderness ratios indicates that wider piles are favourable at higher loads. The results suggest minimised material usage and embodied carbon.

**Figure 11:**
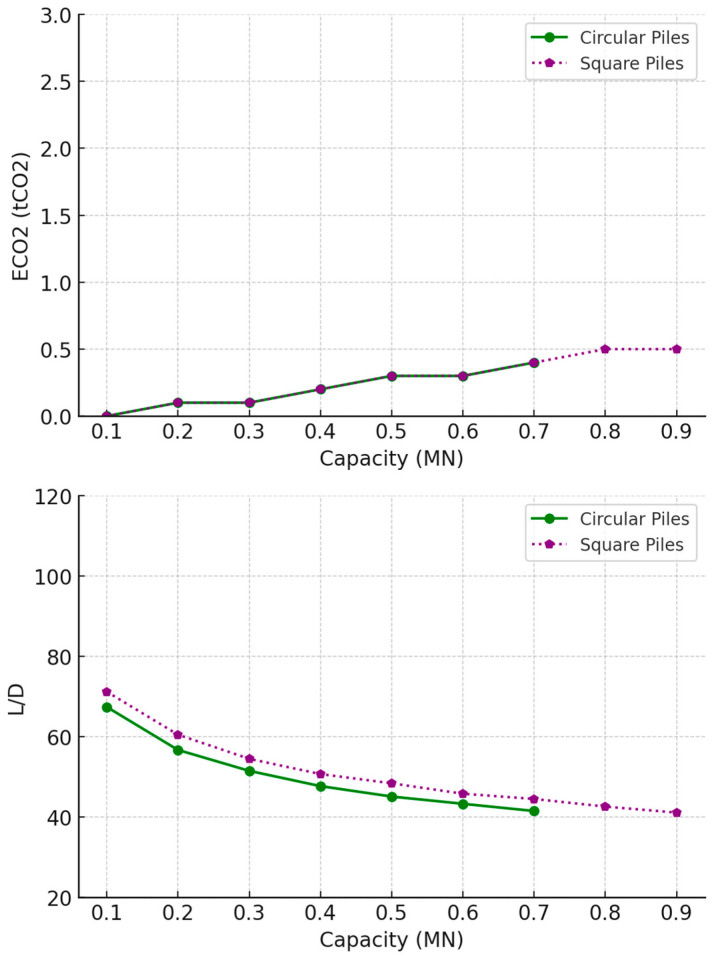
Optimal timber design parameters for tension piles in loose sand soil.

For timber piles, the comparison between circular and square designs in loose sand is shown in Figure 11 and Table 6 and reveals minimal differences in embodied carbon (ECO_2_), with square piles showing a slight advantage in sustainability. In contrast to timber piles in undrained clay, the *L/D* ratios for both pile types decreased consistently with increasing capacity, and the gap between circular and square piles narrowed as capacity approached 0.9 MN. This suggests that in loose sand, both designs performed similarly in terms of geometric efficiency, and the decision between circular and square piles may largely hinge on practical factors such as ease of construction and available resources rather than significant performance differences.

Generally, comparing the results in undrained clay and loose sand indicated significantly lower embodied carbon for the same pile capacities in the case of loose sand. Moreover, in the case of loose sand, concrete and steel piles tended to exhibit steeper declines in their *L/D* ratios, indicating that piles can be shorter relative to their diameter while still achieving the required capacity. This indicates that loose sand offers greater frictional resistance compared with undrained clay, enabling more compact designs. Nevertheless, timber piles are only appropriate for low-capacity applications, typically below 1 MN.

### 3.3. Tension vs. Compression Piles

The overall results of the piling options for tension piles presented in this research were compared with the results of a previous study that investigated the optimal design of compression piles in both undrained clay and loose sand using a similar genetic algorithm [39]. This comparison was used to draw conclusions and show the optimal piling options for different load and soil types.

#### 3.3.1. Tension vs. Compression Piles in Undrained Clay

**Figure 12:**
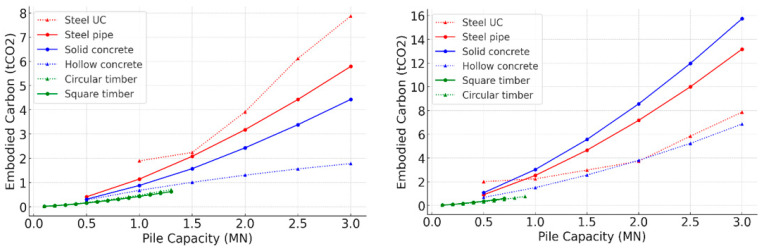
Embodied carbon values for optimal pile design in undrained clay: (**left**) compression piles [39]; (**right**) tension piles.

The comparison of embodied carbon across different pile types for both compression and tension applications revealed significant variations in which options were most sustainable, as demonstrated in Figure 12. Timber piles consistently demonstrated the lowest embodied carbon across both compression and tension applications, making them the most sustainable option. Among the compression piles, timber ranked as the lowest emitting, followed by hollow concrete, which offers a balance of strength and reduced materials use. Solid concrete performed worse due to its greater materials consumption, while steel sections, including pipes and universal columns, had the highest embodied carbon, which is believed to be due to buckling considerations.

For tension piles, timber remains the most sustainable option, exhibiting the lowest embodied carbon. Hollow concrete ranked second, offering a favourable balance between structural performance and carbon impact. Notably, steel UC sections, which performed poorest in compression applications, demonstrated improved efficiency in tension piles, as their performance is primarily governed by the structural properties of the section rather than the volume of material. Solid concrete and steel pipe piles followed, with their higher material demand contributing to increased embodied carbon. As load requirements increase, the disparity between these options becomes more pronounced, emphasising the need for careful selection of pile types to minimise carbon emissions in foundation design.

#### 3.3.2. Tension vs. Compression Piles in Loose Sand

**Figure 13:**
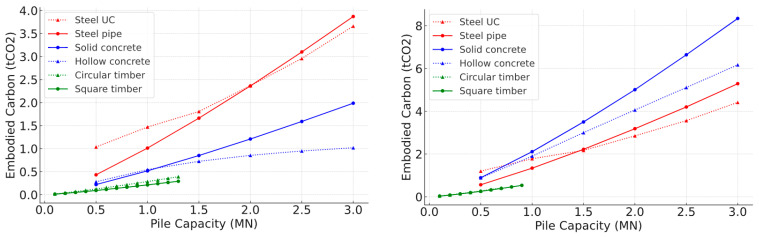
Embodied carbon values for optimal pile design in loose sand: (**left**) compression piles [39]; (**right**) tension piles.

The overall comparison of compression and tension piles designed for use in loose sand is presented in Figure 13. Generally, the highlighted trend of lower embodied carbon in compression piles compared with tension piles prevails. In loose sand, timber piles, particularly square timber, emerged as the most sustainable option for tension piles due to their low embodied carbon. Both timber types maintain low carbon footprints, confirming their efficiency and minimal environmental impact.

Comparing the compression and tension piles’ rankings in loose sand, there appears to be a preference for steel sections as tension piles in granular soil, especially for high load capacities. Steel sections managed to be less emitting than alternative concrete options with the same capacity, due to the negligible base resistance in case of tension piles. This contrasts with the comparison results for fine soil where the steel options were not the favourable design options.

## 4. Case Study

### 4.1. Case Description

**Figure 14:**
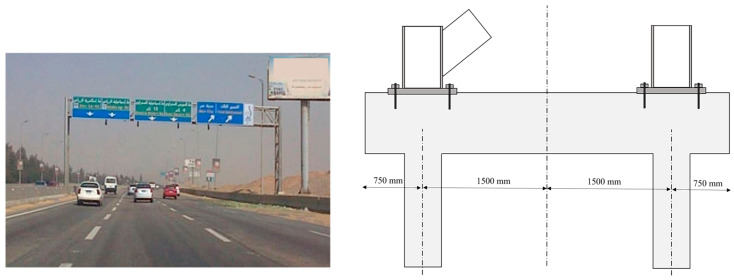
The studied structure and a schematic of the foundation system.

This case study examines the foundation system for an 8 m high crossroad signpost, supported by a double-truss structure. The foundation is composed of a pile cap with two piles, designed to counteract the tension and compression loads resulting from the wind-induced overturning moment on the signpost. The examined design, shown in Figure 14, is a typical off-the-shelf solution provided by a specialist contractor. This arrangement inherently involves horizontal shear forces at the pile head and bending due to the coupled tension–compression forces in the piles. However, it is assumed that tensile forces under wind-induced loading conditions dominate the design considerations. Detailed information on the soil properties and pile design is included as part of the initial setup. The design was optimised using the proposed genetic algorithm, aiming to minimise the embodied carbon while maintaining structural performance. Furthermore, a brief consultation with the contractors is provided to discuss the practicality, feasibility, and potential benefits of the optimised solution compared with the conventional design.

### 4.2. Soil Profile and Pile Design

The soil properties for this case study, as detailed in Table 7, consist of two distinct layers. The first layer is sand and extends from the ground surface to a depth of 25 m, with a unit weight of 19 kN/m^3^. This layer exhibits a deformation modulus ranging from 25 to 50 MPa. The effective friction angle for this layer varies between 32 and 36 degrees, contributing significantly to the pile’s resistance to lateral forces. The second layer, a silty clay found at depths between 25 and 51 m, has a slightly higher unit weight of 20 kN/m^3^ and a lower deformation modulus, between 25 and 30 MPa. This layer also provides some cohesive strength, with an effective cohesion value of 20 to 30 kPa and a relatively lower effective friction angle of 20 degrees.

The original pile design has a diameter of 0.5 m and extends to a depth of 18 m, designed to resist a tension load of 400 kN. The design takes into account the tension forces induced by wind loading on the 8 m signpost. Additionally, the presence of the groundwater table, located at a depth of 8 m, has been factored into the analysis, as it influences both the effective stress within the soil and the pile’s load-bearing behaviour. This combination of soil layers and groundwater conditions makes the foundation design and optimisation more complex and provides a robust case for testing the effectiveness of the proposed genetic algorithm.

### 4.3. Pile Optimisation Results and Discussion

According to the methodology outlined in Section 2.3, the as-built pile design has a tensile resistance of 480 kN and shows an overdesign ratio of 1.2, resulting in a utilisation ratio of 83% and possible potential for 17% pre-optimisation reduction in materials. This utilisation ratio is considered to overcome the effects of uncertainties in soil properties and concrete manufacturing. Also, engineers often design additional spare capacity rather than designing to the limit [63], which is beyond the scope of this research.

**Figure 15:**
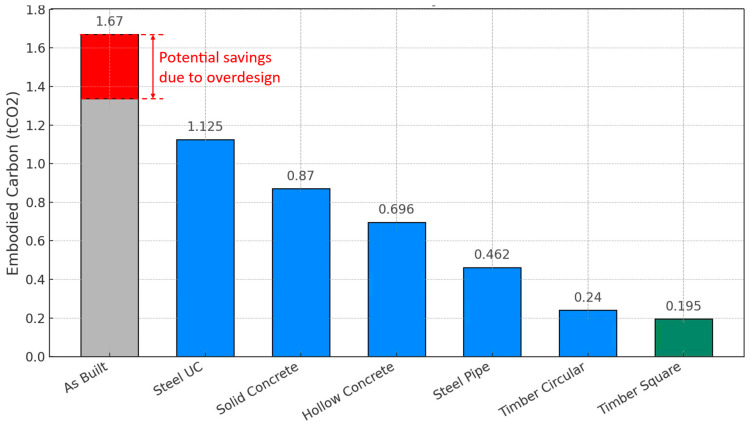
Embodied carbon ranking for the case study’s optimal pile design alternatives.

The average soil properties outlined in Table 7 were utilised as inputs for the optimisation algorithm detailed in Section 2.3 to derive the optimal pile designs for a load-bearing capacity of 480 kN (0.48 MN). The optimisation results, presented in Figure 15 and Table 8, indicate that adopting the optimised designs can lead to significant reductions in embodied carbon. Timber piles were identified as the most sustainable option, with reductions exceeding 88% and 85% for square and circular pile sections, respectively. Steel piles also performed well, offering potential savings of up to 72.3% in terms of embodied carbon compared with the conventional as-built design. Furthermore, concrete piles, both hollow and solid, were able to achieve savings of 58.3% and 47.8%, respectively, despite being essentially the same technology, indicating that optimisation is not being used in practice and thus confirming the importance of this research.

These findings demonstrate the efficacy of the optimisation tool for reducing the embodied carbon associated with pile foundations. However, despite the promising results, several important considerations require further attention, as follows:

- Availability of timber piles: timber piles were shown to be the most sustainable option in this study, especially for lower-capacity pile designs. However, it is crucial to recognise that timber sections are not universally accessible across all regions. In countries with limited forest resources, especially those with arid climates, the availability of piling-grade timber can be a significant limitation [65]. Furthermore, timber prices and logistical challenges associated with transportation and procurement may limit the feasibility of their widespread adoption, especially in regions where alternative materials, such as concrete or steel, are more readily available and economically viable. Another issue remains the unsuitability of timber sections for soils with changing GWT levels, as timber becomes vulnerable to decomposition and loss of strength.
- Industry practices: feedback from contractors reveals that many companies typically adopt off-the-shelf pile designs, with standardised section dimensions and reinforcement specifications. These designs are often conservative to account for the uncertainties associated with varying soil conditions and profiles across different locations. While this approach enhances the robustness of pile designs, it presents a challenge to the adoption of sustainability-focused designs during the early stages of project planning. The industry’s reliance on conservative designs may hinder the transition to more sustainable practices, particularly when optimising for embodied carbon reduction.
- Culture: social factors also play a critical role in the selection of piling solutions. However, social acceptance is difficult to quantify and can vary significantly between regions, clients, and contractors. What is deemed an acceptable or preferable solution in one geographical area or by one contractor may not be viewed similarly elsewhere. This subjectivity adds another layer of complexity when implementing sustainable design options, as local preferences and perceptions can significantly impact decision-making.
- Uncertainties: the embodied carbon of structures varies across different locations due to regional disparities in transportation logistics, material sourcing, and availability. In countries with longer supply chains or limited local resources, higher emissions may result from transporting materials over greater distances, while countries with abundant local materials can reduce embodied carbon significantly. The embodied carbon outcomes presented in this study are sensitive to the emission factors applied, particularly for materials like steel, where variability across product types and production routes is significant. While our primary findings are robust—especially those related to geometric efficiency and optimisation logic—the relative ranking of designs with high steel content may shift under different carbon factor assumptions. We acknowledge this uncertainty and encourage sensitivity testing for project-specific applications.

Generally, the optimisation tool demonstrates considerable potential for reducing the embodied carbon of pile foundations. However, current construction practices, industry habits, and social factors continue to influence the practical implementation of such designs. Further research is necessary to better understand these barriers, and future work by the current authors will focus on addressing the challenges associated with adopting optimised pile designs in real-world construction practices.

## 5. Conclusions

This research presents the first attempt to optimise the embodied carbon in tension piles in different soil types. A hybrid genetic algorithm is proposed to minimise the embodied carbon in tension piles. This algorithm was used to investigate the potential carbon savings of six different pile types: solid and hollow concrete piles, steel pipes, UC, and square and rounded timber sections. The piles were tested and optimised under pure tensile loads up to 3 MN. The optimal design parameters for each pile and soil type were then presented and compared. Finally, a case study assessed the practicality of the proposed optimisation algorithm in minimising the embodied carbon in a future design. The following are the main conclusions:

1. Tension piles in undrained clay were shown to be more emitting than those in loose sand. This is believed to be due to the nature of sandy soil, which exhibits higher surface friction resistance than undrained clay, a main factor that influences the capacity of tension piles.
2. Regarding undrained clay, the optimisation results indicate that while timber piles are restricted to lower capacity applications, square designs exhibit slightly superior environmental performance, though the difference between square and circular sections remains minimal. Hollow concrete piles and UC steel sections demonstrated greater material efficiency and sustainability compared with solid concrete and steel pipe piles, particularly in terms of reducing embodied carbon. The observed efficiency of hollow concrete piles aligns with findings reported in the literature [66].
3. In loose sand, timber piles, though limited to lower capacities, are the most sustainable options for low-capacity piles, with minimal differences in embodied carbon and *L/D* ratios between circular and square designs. Both concrete and steel piles exhibited more compact, material-efficient designs due to steeper declines in their optimal *L/D* ratios compared with the designs for use on undrained clay. Regarding high-capacity piles, UC steel piles outperformed other pile types in terms of embodied carbon.
4. The optimisation tool was applied to an existing case study for a large crossroad signpost and demonstrated significant potential for reducing the embodied carbon in pile foundations, with timber, steel, and hollow concrete piles offering substantial carbon savings. However, practical challenges such as the limited availability of timber, conservative industry practices, and varying social acceptance across regions must be addressed to facilitate the widespread adoption of these sustainable designs in real-world construction.
5. The findings of this study are specific to the selected input soil properties, and variations in soil types may yield different optimal pile designs, as demonstrated in the case study. Nonetheless, the conceptual optimisation technique employed remains applicable across diverse soil conditions, offering flexibility and adaptability for future design scenarios. Designers are therefore encouraged to adapt the proposed optimisation approach to their local contexts and materials data rather than directly relying on the specific findings presented in this paper.

## Figures and Tables

**Table 1:** Soil model properties as tested by Knappet and Craig [49].

Soil Type	Property	Symbol	Value (Unit)
Undrained clay	Unit weight	γ	18 (kN/m^3^)
	Undrained shear strength	*c_u_*	80 + 1.5z * (kPa)
	Poisson’s ratio	*v*	0.2
	Shear modulus	*G*	5000 + 500z * (kPa)
	Adhesion factor	*α*	0.5
Dry loose sand	Unit weight	γ	15 (kN/m^3^)
	Angle of internal friction	*φ′*	32°
	Pile–soil interface angle	*δ*	24°
	Poisson’s ratio	*v*	0.2
	Shear modulus	*G*	10 MPa

* Depth from the top.

**Table 2:** Embodied carbon factors across materials and lifecycle stages [52].

Material	A1–A3 (kgCO_2_e/kg)	A4 (kgCO_2_e/kg)	A5 (kgCO_2_e/kg)	Assumptions
In situ cast concrete	0.082 + 0.002*f_ck_*	0.005 **	0.053	Linear regression of the ICE inventory [15]
Reinforced steel bars	1.99	0.032 *	0.053	Worldwide steel of low recycled content
Construction steel	1.55	0.032 *	0.01	Worldwide open steel sections
Timber	0.263	0.032 *	0.01	Studwork, softwood

* Material considered nationally manufactured with a road travel distance of 300 km [52]. ** Material considered locally manufactured with a road travel distance of 50 km [52].

**Table 3:** Design parameters for the different tested pile geometries.

Sections	Optimised Design Variables	Symbol	Range/Value	Unit
Solid concrete and hollow concrete bored piles	Length	*L_o_*	[1–300]	m
	Outer diameter	*D_o_*	[0.1–3]	m
	Inner diameter (hollow concrete)	*D_i_*	[0.1–3]	m
	Concrete thickness	*t_o_*	[0.1–*D*/2]	m
	Concrete grade	*f_cko_*	[12–60]	MPa
	Reinforcement ratio	*A_s_/A_c_*	[0.004–0.04]	-
Circular and square timber driven piles	Length	*L_o_*	[1–12] *	m
	Diameter or width	*D_o_*	[0.05–0.45]	m
	Timber grade [62]	$f_{ctk}$	Douglas fir C24 $f_{ctk}$ = 18	MPa
Steel pipe and steel universal columndriven piles	Length	*L_o_*	[1–300]	m
	Section diameter/height	*D_o_*	[0.1–3]	m
	Pipe or web thickness	*t_o_*	[0.001–0.05] [63]	m
	Flange width	*b_o_*	[0.15–0.48]	m
	Steel grade	$f_{y}$	S355 $f_{y}$ = 355	MPa

* Mechanical couplers are used if length exceeds 12 m.

**Table 4:** Optimal design parameters for concrete piles at different load capacities.

Capacity (MN)	Clayey Soil					Sandy Soil				
	Solid		Hollow			Solid		Hollow		
	*L_o_* (m)	*D_o_* (m)	*L_o_* (m)	*D_o_* (m)	*D_i_* (m)	*L_o_* (m)	*D_o_* (m)	*L_o_* (m)	*D_o_* (m)	*D_i_* (m)
0.5	18.5	0.6	4.2	1.6	1.4	21.1	0.25	19.0	0.4	0.2
1	26.2	0.6	10.0	2.0	1.8	25.1	0.3	19.8	0.6	0.4
1.5	32.1	0.6	13.4	2.0	1.8	27.8	0.4	19.3	0.9	0.7
2	37.0	0.7	5.0	2.0	1.8	29.9	0.5	19.0	1.2	1.0
2.5	41.4	0.8	16.7	2.0	1.8	31.6	0.5	18.8	1.5	1.3
3	45.3	0.9	20.0	2.0	1.8	33.1	0.6	18.7	1.9	1.7

**Table 5:** Optimal design parameters for steel piles at different load capacities.

(MN)	Clayey Soil					Sandy Soil				
	Pipe Section			UC Section		Pipe Section			UC Section	
	*L_o_* (m)	*D_o_* (m)	*t_o_* (mm)	*L_o_* (m)	Section	*L_o_* (m)	*D_o_* (m)	*t_o_* (mm)	*L_o_* (m)	Section
0.5	39.8	0.19	4.0	30.2	152 × 152 × 37	25	0.19	4.0	21.8	152 × 152 × 23
1	50.3	0.22	5.0	32.0	152 × 152 × 51	29.7	0.22	5.0	25.4	152 × 152 × 23
1.5	68.9	0.32	6.0	41.0	203 × 203 × 46	32.9	0.32	6.0	31.0	203 × 203 × 46
2	79.6	0.36	8.0	41.3	203 × 203 × 52	36.4	0.36	8.0	31.7	203 × 203 × 100
2.5	89.0	0.41	8.0	51.6	254 × 254 × 73	37.4	0.46	8.0	31.9	257 × 254 × 167
3	97.4	0.46	8.0	58.0	305 × 305 × 79	39.1	0.46	10	32.7	305 × 305 × 18

**Table 6:** Optimal design parameters for timber piles at different load capacities.

Capacity (MN)	Clayey Soil				Sandy Soil			
	Round Pile		Square Pile		Round Pile		Square Pile	
	*L_o_* (m)	*D_o_* (m)	*L_o_* (m)	*D_o_* (m)	*L_o_* (m)	*D_o_* (m)	*L_o_* (m)	*D_o_* (m)
0.1	9.8	0.17	8.7	0.15	11.6	0.17	10.7	0.15
0.2	13.9	0.24	12.3	0.21	13.5	0.24	12.7	0.21
0.3	17.1	0.29	15.1	0.26	14.9	0.29	14.1	0.26
0.4	19.7	0.34	17.4	0.30	16.1	0.34	15.1	0.30
0.5	22.0	0.38	19.5	0.33	17.0	0.38	16.0	0.33
0.6	24.1	0.41	21.4	0.37	17.8	0.41	1.7	0.37
0.7	26.0	0.45	23.1	0.40	18.9	0.45	17.4	0.39
0.8	-	-	24.7	0.42	-	-	18.0	0.42
0.9	-	-	26.2	0.45	-	-	18.0	0.45

**Table 7:** Soil properties in the case study.

Soil Parameter [Symbol] (Unit)	Layer 1—Sand	Layer 2—Silty Clay
Layer depth [*z*] (m)	[0–25]	[25–42]
Unit weight [*γ*] (kN/m^3^)	19	20
Young’s modulus [*E*] (MPa)	[25–50]	[25–30]
Effective cohesion [*c*] (kPa)	0	[20–30]
Effective friction angle [*φ*] (Degrees)	[32–36]	20

**Table 8:** Optimisation results for the case study tension pile with a capacity of 400 kN.

Pile Type	Design	Embodied Carbon
As-built design	*L* = 18 m *D* = 0.5 m *f_ck_* = 25 MPa *A_s_/A_c_* = 1.5%	Reference value
Solid concrete	*L* = 20 m *D* = 0.25 m *f_ck_* = 25 MPa *A_s_/A_c_* = 4%	−47.9%
Hollow concrete	*L* = 17 m *D_o_* = 0.3 m *D_i_* = 0.1 *f_ck_* = 25 MPa *A_s_/A_c_* = 2.4%	−58.3%
Steel pipe	*L* = 23.62 *D* = 0.2 m *t* = 3 mm	−72.3%
Steel UC	*L* = 16 m Section = 203 × 203 × 46	−32.6%
Circular timber	*L* = 16.5 *D* = 0.35 Douglas fir C24	−85.6%
Square timber	*L* = 15.5 *D* = 0.3 Douglas fir C24	−88.3%

## Data Availability

The original contributions presented in this study are included in the article. Further inquiries can be directed to the corresponding author.
